# Financing of physical rehabilitation services in Iran: a stakeholder and social network analysis

**DOI:** 10.1186/s12913-020-05447-4

**Published:** 2020-07-01

**Authors:** Saeed Shahabi, Ahmad Ahmadi Teymourlouy, Hosein Shabaninejad, Mohammad Kamali, Kamran Bagheri Lankarani

**Affiliations:** 1 Health Policy Research Center, Institute of Health, Shiraz University of Medical Sciences, Shiraz, Iran; 2 Department of Health Services Management, School of Health Management and Information Sciences, Iran University of Medical Sciences, Tehran, Iran; 3 Population Health Sciences Institute, Newcastle University, Newcastle upon Tyne, UK; 4 Rehabilitation Research Center, Department of Rehabilitation Management, School of Rehabilitation Sciences, Iran University of Medical Sciences, Tehran, Iran

**Keywords:** Physical rehabilitation, Financing, Stakeholder analysis, Social network analysis

## Abstract

**Background:**

Inadequate financing is one of the major barriers in securing equitable access to high-quality physical rehabilitation services, without imposing financial hardship. Despite this, no sufficient attention has been paid to physical rehabilitation services and no specific financial resources have been allocated to such services in many countries including Iran. Owing to the fact that effective decision- and policy-making requires identifying possible stakeholders and actors and their characteristics, in the current study a stakeholder analysis and also a social network analysis (SNA) was conducted to identify the potential stakeholders and also their characteristics involved in physical rehabilitation financing (PRF)-related policies in Iran.

**Methods:**

The present study was performed in two phases. Firstly, semi-structured interviews and relevant document review were conducted to identify the stakeholders. Then, the position, power, interest, and influence of each stakeholder were determined using a web-based questionnaire. Secondly, SNA approach was utilized to map and visualize the interactions among stakeholders.

**Results:**

The findings showed that there are different stakeholders in PRF-related decision- and policy-making processes in Iran. In addition, the position, power, interest, and influence level of the identified stakeholders were varied. Moreover, although some stakeholders, like the Ministry of Health and the parliament have the highest level of power and position, they lack sufficient interest to participate in PRF-policies. Furthermore, SNA demonstrated that social network density was low, which indicates the lack of proper collaboration and interaction among the stakeholders.

**Conclusion:**

As many powerful and influential stakeholders had low interest levels to warrant participate in the FPR-related decision- and policy-making processes in Iran, employing careful and effective strategies, that is ongoing negotiations, receiving advocacy, and making senior managers and policy-makers aware can be helpful.

## Background

Globally, people with disability account for approximately 15% of the population [1], and with the increasing prevalence of chronic conditions, musculoskeletal disorders, aging, and traffic injuries as well as the increase in the survival rates following trauma and other severe diseases, the need for more rehabilitation services has become ever more crucial [2–4]. Furthermore, Sustainable Development Goal 3.8 is encouraging countries worldwide to secure equitable access to high-quality health care services, including rehabilitation without imposing financial hardship. Indeed, universal rehabilitation coverage is a crucial part of universal health coverage [5].

Rehabilitation services including physical rehabilitation services (e.g. physiotherapy, occupational therapy, prosthetics, and orthotics services) are considered as a group of interventions to reduce the functional and physical limitations in order to obtain a maximum level of independence [6]. As a result, these services may have significant advantages for individuals, society, and also national economies [7–9]. Additionally, physical rehabilitation services also have preventive effects [10, 11] and facilitate the hospital discharge process [12, 13]. Therefore, not only does the provision of affordable physical rehabilitation services not impose any significant financial pressure on the health sector, but also it can be a rational investment [14–16].

Despite the above-mentioned points, no sufficient attention has been paid to physical rehabilitation services, especially in developing counties like Iran, in such a way that a large proportion of service users face financial hardship [14, 17]. In response to this situation, the World Health Organization (WHO) and other international organizations have been involved in many activities in this respect, in the past few decades [16]. Recently, the ‘WHO Global Disability Action Plan 2014-2021’, ‘Rehabilitation 2030; a call for action’ and ‘Rehabilitation in Health Systems’ were released by the WHO aiming to promote and strengthen rehabilitation services in the health sector [18–20]. Although enhancing the financing for rehabilitation by adopting appropriate mechanisms has been emphasized, the evidence shows catastrophic health expenditure (CHE) and high rate of out-of-pocket (OOP) payments when receiving physical rehabilitation services [21]. In fact, no specific financial resources were allocated to physical rehabilitation services in many countries like Iran [14]. Traffic injuries, 8-year of imposed war, climate changes, aging, natural disasters, and chronic diseases are the main causes of disability, leading to the increased need for utilizing these services in Iran. Since Iran is one of the top-ten traffic-accident prone countries in the world, head trauma, fractures, and spinal cord injuries are the most important causes of disability incidence in this country. Furthermore, improved health status in Iran resulted in a considerable move towards aging population. Therefore, well-planned, coordinated, and careful financing policies are needed to ensure that physical rehabilitation services are covered and provided for by the best financial risk protection strategies [22].

Effective decision- and policy-making requires identifying potential stakeholders and actors and their characteristics [23]. Stakeholders are actors (organizations or individuals) that may affect a policy [24], and stakeholder analysis is a process of identifying and understanding the characteristics of such actors by evaluating their position, power, interest, and influence on policy-making [25]. Based on the current literature, there are a variety of stakeholders in the health care physical rehabilitation sectors in Iran, who can affect physical rehabilitation financing (PRF)-related policies [15, 21]. Therefore, recognizing the relevant stakeholders and their relationships is one of the main steps in developing and implementing PRF-policies.

The aim of this study was to identify the potential stakeholders and also their characteristics involved in PRF-related policies in Iran in order to pave the way for developing appropriate policies and practical strategies to improve the financing of physical rehabilitation in Iran.

## Methods

**Fig. 1:**
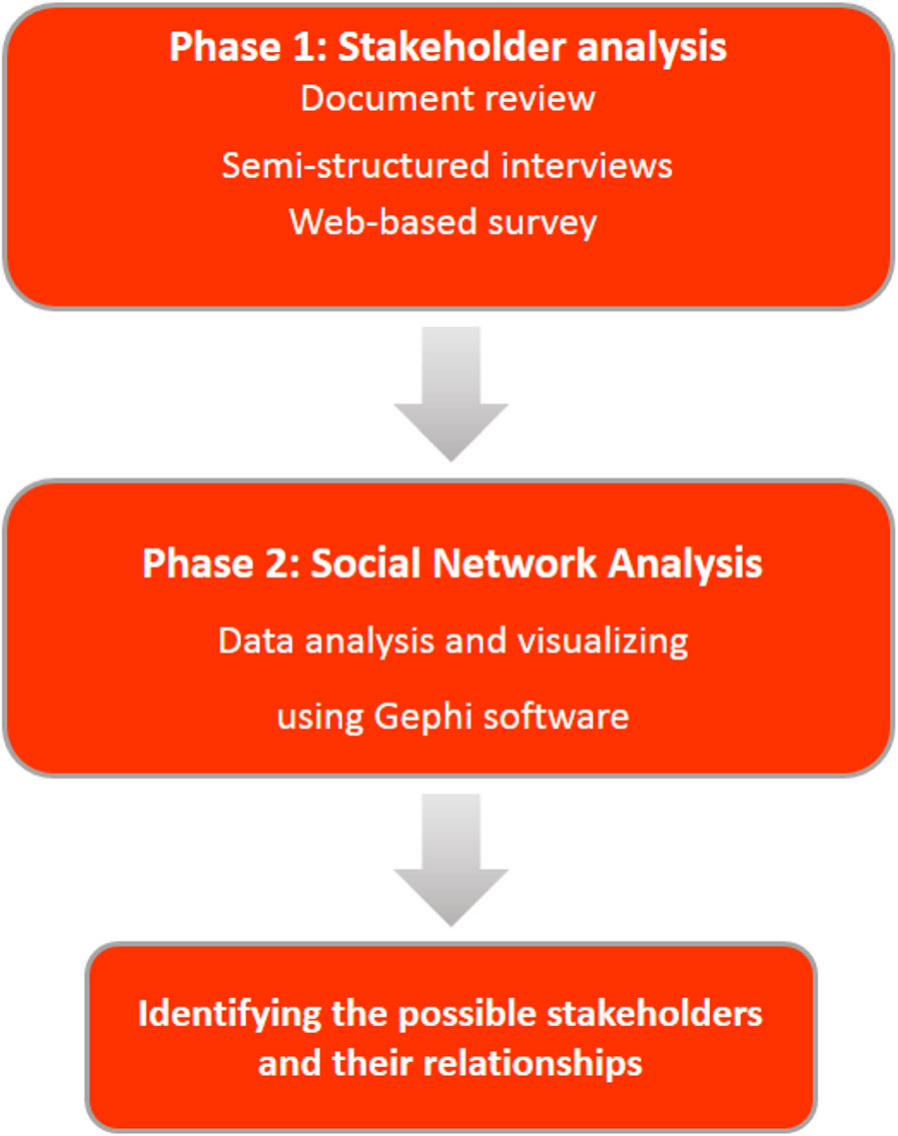
Overview of study methods

This study was conducted in two phases. Firstly, a qualitative content analysis was performed to identify the relevant stakeholders and, secondly, Social Network Analysis (SNA) was used to understand the relationships and interactions between different stakeholders. An overview of the study methods has shown in Fig. 1.

### Phase Ι: stakeholder analysis

#### First step

##### Sampling strategy

To identify the relevant stakeholders involved in the financing of physical rehabilitation services in Iran, individual semi-structured interviews were conducted. The participants were recruited using purposive and snowball sampling. During sampling, maximum variation in terms of gender, scientific background, employment status, and executive experience was considered. The selection process continued until data saturation was achieved. In total, we conducted 36 semi-structured interviews to find the relevant stakeholders in financing of physical rehabilitation services in Iran. The duration of each interview varied from 15 to 20 min.

##### Data collection techniques

Semi-structured interviews were conducted with key informants in health financing and physical rehabilitation sectors (Table 1) by the first author (a male PhD Health Policy-maker) from January 2019 to September 2019. The main question raised was; “Who are the main physical rehabilitation financing-related actors (including institutions and organizations) involved in the decision- and policy-making processes in Iran?” Prior to interviews, a written informed consent form including ethical principles and study aims was sent by the first author (via official and personal email) to the participants. Additionally, if the participant had not signed the form electronically, the interviewer re-read the contents of the consent form verbally at the beginning of face-to-face sessions and received the signed form.

**Table 1:** List of participants

Participants	No.
Health policy-maker	6
Rehabilitation policy-maker	4
Physiotherapist	6
Orthotist	5
Prosthetist	3
Occupational therapist	4
Faculty member	8

##### Data analysis

All interviews were transcribed verbatim by the first author. The anonymized transcripts were examined using thematic content analysis in accordance with the Braun and Clarke approach by three authors (SSH, AA, and MK).

##### Rigor and trustworthiness

Based on the Guba and Lincoln approach, credibility, confirmability, dependability, transferability, and authenticity criteria were considered to improve the rigor and trustworthiness of findings [26]. Hence, peer debriefing (credibility), member-checking by contributors (confirmability), participation of several authors in analysis process (dependability), applying maximal variation sampling (transferability), and using citations from almost all individuals (authenticity), were adopted through the study.

Then, relevant policy documents, including documents and reports developed and implemented by the government, the parliament (in Persian *Majles*), the High Council of Health Insurance (HCHI), the Social Security Organization, the Iran Health Insurance Organization (IHIO), the Armed Forces Social Security Organization (AFSSO), the State Welfare Organization of Iran, scientific associations (Iranian Physiotherapy Association, The Iranian Scientific Association for Orthotics & Prosthetics, and Iranian Occupational Therapy Association), the Foundation of Martyrs and Veterans Affairs (FMVA), the Red Crescent (in Persian *Hilal Ahmar*), the WHO and Eastern Mediterranean Region Organization (EMRO) reports, and Scholarly literature, were considered to discover other potential actors. In total, the research team prepared a list of relevant actors and stakeholders based on the interviews and document analysis.

#### Second step

According to the theoretical framework adopted in previous studies [25], four items (namely position, power, interest, and influence) were chosen to analysis the identified stakeholders. Subsequently, an online web-based questionnaire was designed in which stakeholders were ranked with respect to position, power, interest, and influence in PRF-related policies and plans. Position was defined as relationship and interaction of one stakeholder with other stakeholders involved in the decision- and policy-making process; power was defined as the ability to and extent to which decision- and policy-making processes may be affected by them; influence was defined as the amount of available and potential resources (including knowledge, money, and facilities); and interest was defined as the level and extent of participation in the decision- and policy-making process. The link of the online questionnaire was posted via E-mail or WhatsApp to the interviewees. Participants were asked to express their viewpoints for each item using a five-point rating scale (low, medium-low, medium, medium-high, and high). All participants completed and submitted their responses. However, only one participant’s response was incomplete and the research team decided to remove it. The final value of participants for each stakeholder was calculated based on the geometric mean.

### Phase ΙΙ: social network analysis (SNA)

SNA represents various special methodologies to measure, analyze, and map the social interactions and relationships among different actors, organizations, and teams [27]. It facilitates the investigation of types and patterns of collaboration among stakeholders, where these stakeholders are visually presented in a map by nodes, and interactions between these nodes [28]. Indeed, this network analysis can be a distinctive approach to recognize the structure of decision- and policy-making processes and also interaction between potential actors [29]. Therefore, using SNA can facilitate the identification of the relevant parts that need to be negotiated and addressed, with the aim of improving the health related policy-making processes and obtaining appropriate interactions among the involved stakeholders [25, 30]. SNA uses several metrics to describe the network, which can be categorized into two groups including overall structure (e.g. density and diameter) and individual actor metrics (degree, betweenness, closeness, and eigenvector centralities) [31].

Concisely, the network density defines a measure of network cohesion, and diameter gives an idea of the length of the extended geodesics of the network [31]. Centrality measures try to determine the stakeholders and discover those who have central roles in the network [32]. Degree centrality is the number of edges (direct relationships) that a stakeholder has. Stakeholders with higher degree centrality are introduced as very visible and have more influence on their neighborhood. Closeness centrality is achieved with the average length of the paths linking the stakeholder to others. This centrality reveals the reachability of a stakeholder to others in the social network. Betweenness centrality calculates the capacity of a stakeholder to be an intermediator between any two nodes. Stakeholders with high betweenness centrality, have a strategic role. Finally, eigenvector centrality demonstrates the importance and influence of a stakeholder in accordance with its connections with central actors [25, 31, 33]. To conduct the SNA, collected data were organized in Excel Template and then analyzed and visualized using the Gephi version 0.9.2 software [34].

## Results

### Stakeholder analysis

Several stakeholders were identified including the Elites, the FMVA (which finance and provide rehabilitation services to war veterans), the Governmental insurers (including a major part of the health insurance market), the Judicial system (as a public claimant it can assist in enforcing laws related to rehabilitation), the Mass media, the Medical universities, the Ministry of Health and Medical education (MOHME, involved in related decision- and policy-making processes), the Non-governmental organizations (NGOs, which play a significant role in financing and providing rehabilitation services especially for the poor and disabled groups), the Parliament (involved in enacting and overseeing law enforcement), the Physicians, the Planning and Budget Organization (PBO, as a budget regulator in various areas including health and welfare sectors), the Private insurers, the Providers (Physiotherapists, Occupational therapists, Prosthetists, and Orthotists), the Public, the Red Crescent (involved in financing and providing of physical rehabilitation services), the Research centers, the Scientific associations (involved in regulatory and therapeutic guidelines), the United Nations (UN), the State Welfare Organization, the EMRO, and the WHO. The identified stakeholders participated in different steps of related decision- and policy-making process, from agenda-setting to policy implementation and evaluation.

After sending the web-based questionnaire, all participants responded. Finally, the data of the 35 questionnaires were analyzed. Table 2 demonstrates the estimated position, power, interest, and influence of the stakeholders involved in the financing of physical rehabilitation related decision- and policy-making processes.

**Table 2:** Rating the stakeholders according to position, power, interest, and influence

Stakeholders	Position	Power	Interest	Influence
Users	Low-medium	Low	Medium-high	Low
Elites	Low-medium	Medium	Medium-high	Low-medium
EMRO	Low-medium	Medium	Medium	Medium
Foundation of Martyrs and Veterans Affairs	Medium	Medium-high	Medium-high	Medium-high
Governmental insurers	Medium-high	Medium-high	Low	Medium-high
Juridical system	Medium-high	High	Low-medium	Medium-high
Mass media	Medium-high	Medium-high	Low-medium	Medium-high
Medical universities	Medium	Low-medium	Medium	Low-medium
Ministry of Health and Medical Education	Medium-high	High	Low	High
Non-governmental organizations (NGOs)	Low-medium	Low-medium	Medium-high	Low-medium
Parliament	High	High	Low-medium	High
Physicians	Medium	Medium-high	Low-medium	Medium
Planning and Budget Organization	Medium	Medium-high	Low-medium	Medium-high
Private insurers	Medium	Medium	Low	Medium
Providers	Low-medium	Medium	Medium	Medium
Public	Low-medium	Medium	Medium	Low-medium
Red Crescent	Low-medium	Medium	Medium-high	Medium
Research centers	Low-medium	Low-medium	Medium-high	Low-medium
Scientific associations	Low-medium	Low	Medium-high	Low
UN	Medium-high	Medium	Medium	Medium
Welfare Organization	Medium-high	Medium	High	Medium-high
WHO	Medium-high	Medium	High	Medium-high

### Position analysis

Participants determined the parliament as the highest stakeholder in terms of position. Indeed, parliament can play a considerable role in financing physical rehabilitation in accordance with its facilities and also duties. Notably, governmental insurers (including SSO, IHIO, and AFSSO) were rated medium-high level. In fact, a major proportion of funding for the health sector comes from these insurers. Furthermore, the State Welfare Organization, judicial system, the MOHME, and mass media were recognized at medium-high level in terms of position. In regard to this item, international stakeholders (including the WHO and UN) were found to rank medium-high. FMVA, PBO, private insurers, physicians, as well as medical universities were rated medium. Summarily, various stakeholders e.g. users, providers, public, and Red Crescent had low-medium level in terms of position.

### Power analysis

Among the identified stakeholders, the MOHME, the parliament, and the judicial system had the highest power level towards PRF-related policy-making processes in Iran. Additionally, PBO, physicians, mass media, governmental insurers, and FMVA were rated medium-high. Surprisingly, the State Welfare Organization and Red Crescent had medium power level, whereas these stakeholders play a significant role in the physical rehabilitation sector. In general, stakeholders like medical universities, NGOs, and research centers were rated low-medium in terms of power. However, users and scientific associations were rated as low.

### Interest analysis

Based on the findings, the State Welfare Organization and also the WHO had the highest interest level towards PRF-related policies, providing numerous recommendations and involved in a great many activities in this domain. In addition, users (clients), elites, FMVA, and NGOs as well as the Red Crescent, research centers, and scientific associations have been recognized at medium-high level in terms of the interest item. Participants also reported that a number of stakeholders have medium interest to participate in PRF-related policies, such as medical universities, providers, the public, EMRO, and UN. In addition, judicial system, mass media, parliament, PBO, and physicians were rated at low-medium level. However, the MOHME, governmental insurers, and Private insurers had the lowest rate. Indeed, these stakeholders were not interested in participating in PRF-related policy-making processes.

### Influence analysis

Participants stated that the MOHME and the parliament were the highest stakeholders in terms of influence. In addition, FMVA, governmental insurers, judicial system, mass media, PBO, the State Welfare Organization, and the WHO had a medium-high influence on the PRF-related policies in Iran. In fact, these stakeholders play an important role in health and welfare financing processes including physical rehabilitation services. EMRO, UN, physicians, private insurers, providers, and the Red Crescent were rated medium, whereas scientific elites, medical universities, NGOs, the Public, and research centers were determined as stakeholders with low-medium influence in PRF-related policies. In total, users and scientific associations were rated at low level in influence item.

### Social network analysis (SNA)

**Fig. 2:**
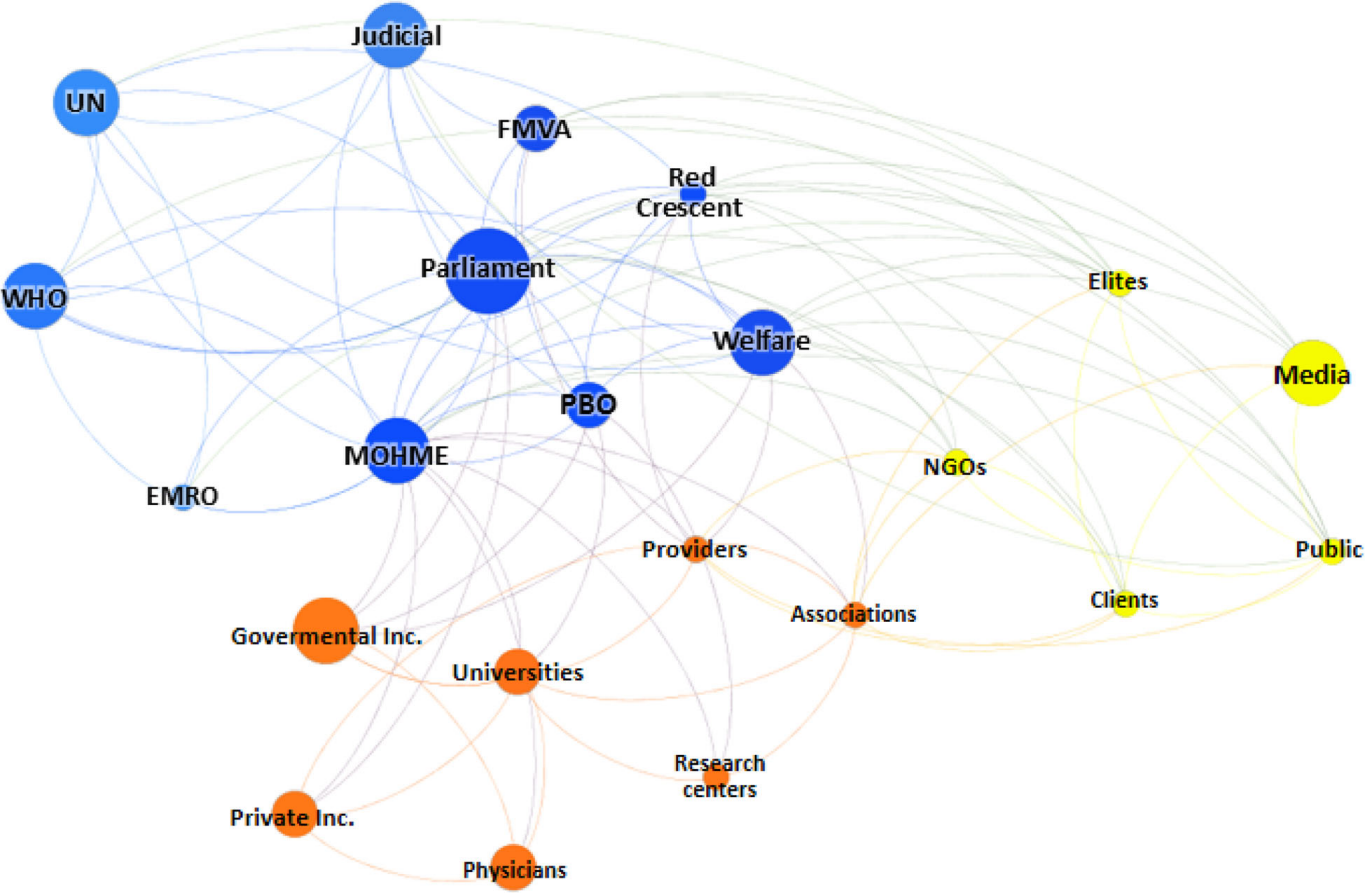
Network map based on the position of stakeholders. Abbreviations: UN: the United Nations; WHO: World Health Organization; EMRO: Eastern Mediterranean Region Organization; FMVA: Foundation of Martyrs and Veterans Affairs; MOHME: Minstry of Health and Medical Education; Inc.: Insurances; PBO: Planning and Budget Organization; Welfare: the State Welfare Organization; NGOs: Non-Governmental Organizations

**Fig. 3:**
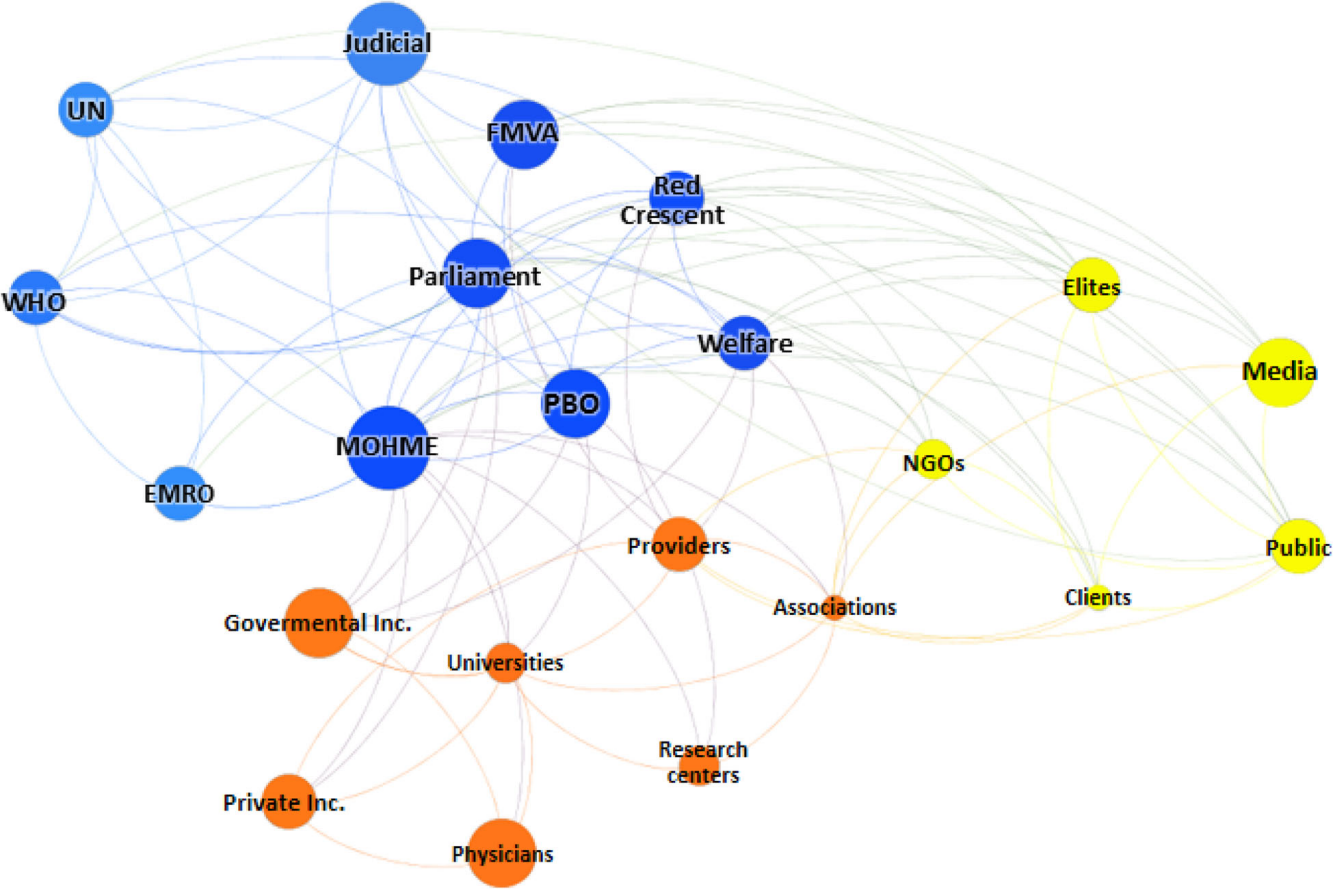
Network map based on the power of stakeholders. Abbreviations: UN: the United Nations; WHO: World Health Organization; EMRO: Eastern Mediterranean Region Organization; FMVA: Foundation of Martyrs and Veterans Affairs; MOHME: Minstry of Health and Medical Education; Inc.: Insurances; PBO: Planning and Budget Organization; Welfare: the State Welfare Organization; NGOs: Non-Governmental Organizations

**Fig. 4:**
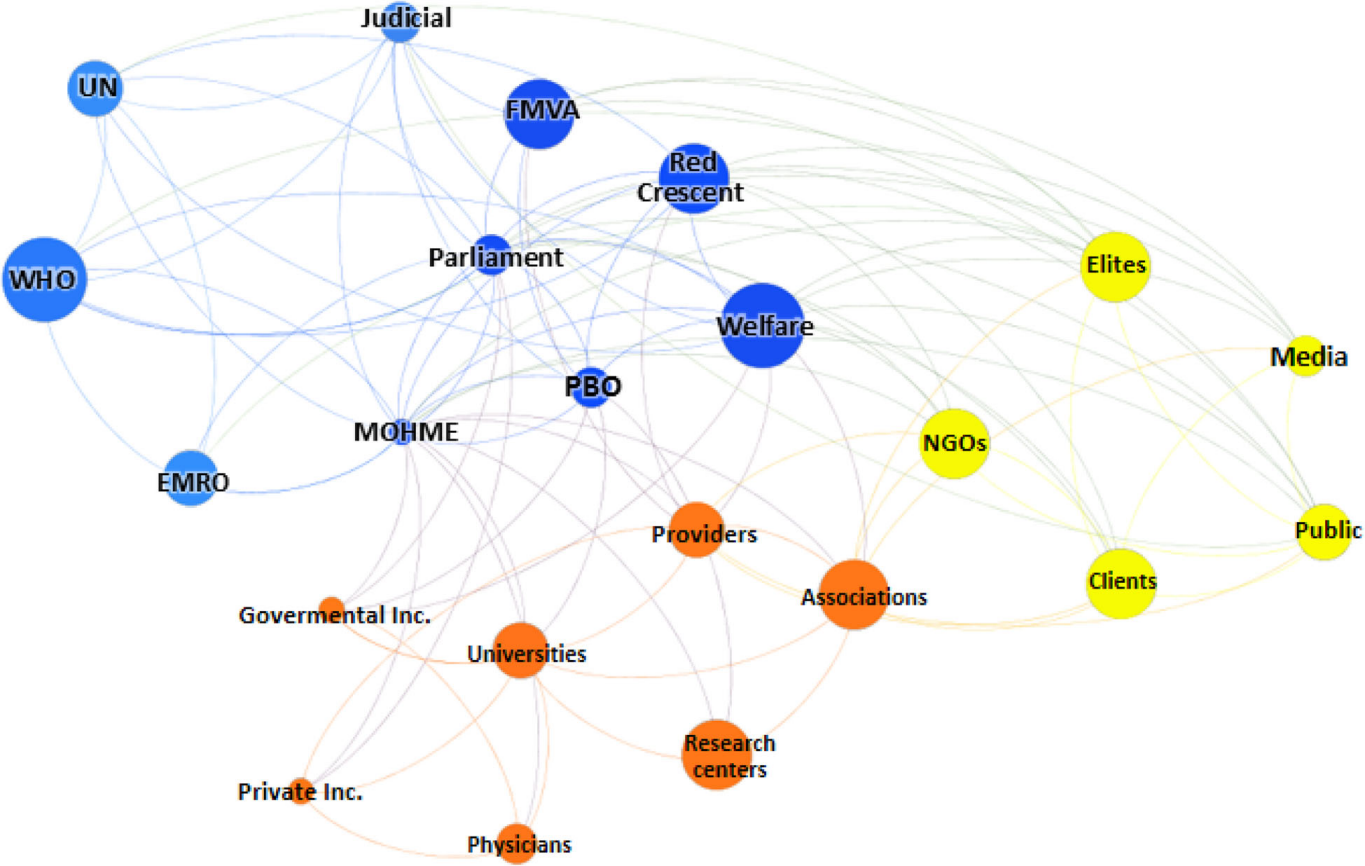
Network map based on the interest of stakeholders. Abbreviations: UN: the United Nations; WHO: World Health Organization; EMRO: Eastern Mediterranean Region Organization; FMVA: Foundation of Martyrs and Veterans Affairs; MOHME: Minstry of Health and Medical Education; Inc.: Insurances; PBO: Planning and Budget Organization; Welfare: the State Welfare Organization; NGOs: Non-Governmental Organizations

**Fig. 5:**
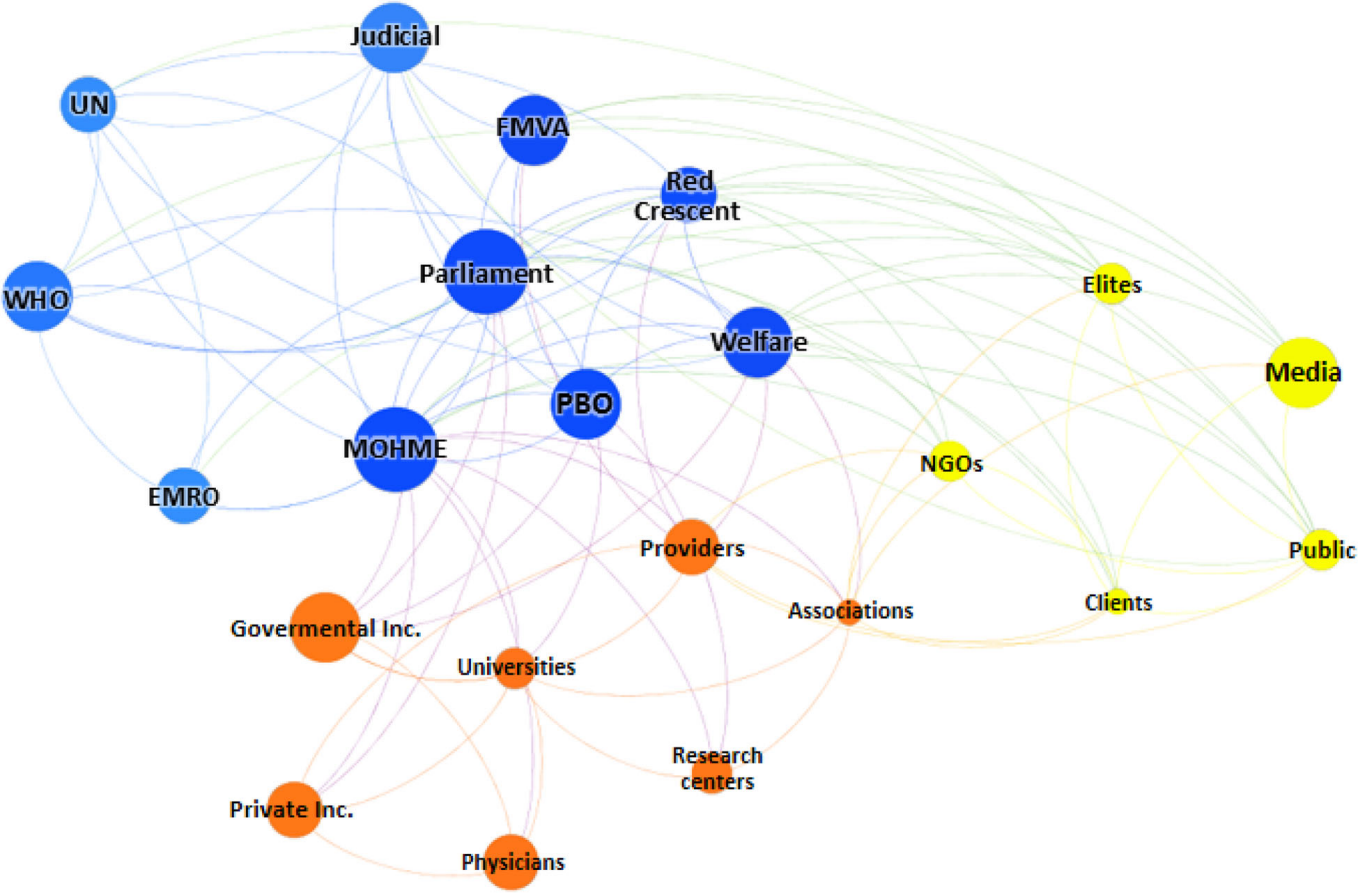
Network map based on the influence of stakeholders. Abbreviations: UN: the United Nations; WHO: World Health Organization; EMRO: Eastern Mediterranean Region Organization; FMVA: Foundation of Martyrs and Veterans Affairs; MOHME: Minstry of Health and Medical Education; Inc.: Insurances; PBO: Planning and Budget Organization; Welfare: the State Welfare Organization; NGOs: Non-Governmental Organizations

The calculated social network metrics including degree centrality, closeness centrality, betweenness centrality, eigenvector centrality, hub, and page rank are showed in Table 3. Furthermore, network and node-level metrics including nodes, edges, density, diameter, and so on are reported in Table 4. As shown, the MOHME, the parliament, and the State Welfare Organization had the highest rank for PRF-related decision- and policy-making processes in Iran. In accordance with the SNA findings, the public, the Red Crescent, judicial system, and elites were the next in rank. Figures 2, 3, 4, and 5 are showed the network maps based on position, power, interest, and influence of the involved stakeholders in PRF-related decision- and policy-making in Iran.

**Table 3:** Social network metrics

Stakeholders	Degree centrality	Closeness centrality	Betweenness centrality	Eigenvector centrality	Hub	PageRank
Ministry of Health and Medical Education	21	0.87	46.74	1.00	0.38	0.074
Parliament	18	0.80	23.24	0.92	0.36	0.065
Welfare Organization	15	0.77	12.69	0.78	0.30	0.061
Public	11	0.67	3.97	0.61	0.24	0.046
Red Crescent	11	0.67	4.75	0.62	0.24	0.046
Juridical system	10	0.61	1.68	0.53	0.19	0.034
Elites	10	0.65	5.09	0.56	0.22	0.042
Planning and Budget Organization	10	0.63	6.75	0.57	0.22	0.039
Providers	10	0.65	7.20	0.50	0.19	0.044
Medical universities	10	0.61	4.99	0.36	0.12	0.039
Scientific associations	9	0.63	7.64	0.44	0.17	0.041
Applicants	9	0.61	1.53	0.49	0.19	0.040
WHO	9	0.61	0.99	0.57	0.22	0.034
UN	8	0.61	0.99	0.49	0.19	0.033
Non-governmental organizations	8	0.61	0.74	0.49	0.19	0.035
Mass media	7	0.58	1.32	0.39	0.15	0.032
Foundation of Martyrs and Veterans Affairs	7	0.58	2.01	0.37	0.14	0.031
Governmental insurers	6	0.58	1.87	0.35	0.13	0.028
EMRO	6	0.56	0.00	0.41	0.16	0.023
Private insurers	5	0.56	1.27	0.27	0.10	0.025
Research centers	4	0.55	0.20	0.22	0.08	0.020
Physicians	4	0.51	0.25	0.18	0.07	0.022

**Table 4:** Network and node-level metrics

Parameter	Value
Nodes	22
Edges	105
Density	0.45
Average clustering coefficient	0.64
Average degree	9.45
Number of triangles	516
Diameter	3
Average path length	1.58

## Discussion

The current study showed that there are different stakeholders in PRF-related decision- and policy-making processes in Iran. In addition, the position, power, interest, and influence level of the identified stakeholders were varied. Recognizing the important stakeholders and also their roles can be considered a necessary step to develop, adopt, and implement effective policy solutions [24, 35]. Managers and policy-makers may also use the findings of the stakeholder analysis in open discussions to achieve consensus [24]. In regard to the PRF-related policies in Iran, although some stakeholders like MOHME and parliament have the highest level of power and position, they do not have adequate interest to participate.

Although the financing of physical rehabilitation services is identified as a challenge for the Iranian health care system [21], lack of interaction and corporation has always been a main barrier to policy formulation and implementation [15]. Our results in the present investigation also confirmed this phenomenon. Therefore, using negotiations can be an essential strategy to mitigate current dissents, and facilitate moving to optimal compromises [36]. Unfortunately, some participants believed that the MOHME did not hold itself responsible for financing and providing physical rehabilitation services in Iran, although these services are introduced as a part of the health system [16].

One of the main factors affecting the decision- and policy-making is the power of stakeholders [37]. However, regarding PRF-related policies, powerful stakeholders have little interest in participation [14]. This is a considerable challenge that prevents relevant topics from being included in the agenda setting [38]. Consequently, various strategies such as informing the key stakeholders about physical rehabilitation services and the growing demand for them [15], must be applied to increase the chances of putting these issues on the agenda [39].

In regard to position, high ranking stakeholders like parliament, may have a significant effect on other actors [25]. In fact, contextual features of each actor represents its role in policy-making processes. Organizing a policy dialogue with senior legislators and policy-makers can be a rational way to deliberate on this issue, discover policy options to address it, and employ effective implementation strategies [40]. Indeed, as a study in Nigeria demonstrates [41], policy dialogues can improve evidence-to-policy relationship.

According to the available evidence, hybrid theories, including top-down and bottom-up approaches, must be considered to facilitate the policy implementation [42, 43]. However, top-down approach is more prevalent in Iran, especially in the health care system [14, 15], so that central stakeholders with high level of power, position, and influence, make decisions without the involvement of low-level actors such as disabled persons, therapists, scientific associations and so on [15]. For instance, the focus of the Iranian health transformation plan on the financing of treatment interventions and lack of funding for physical rehabilitation services illustrates this situation [44, 45]. Therefore, strengthening related NGOs, increasing the involvement of rehabilitation professionals, and also participation of disabled persons in policy-making processes can be possible solutions [46, 47]. For instance, NGOs are use as advisors and technical supporters during policy-making in Senegal and Mozambique to promote the rehabilitation services [48].

Stakeholders have a varied spectrum of influence in accordance with their interests and attitudes [49]. Our findings showed that the MOHME and the parliament have highest influence on PRF-related polices. However, despite this high influence level, because of political, economic, and social issues, these stakeholders do not prioritize the financing of physical rehabilitation services, and mainly focus on services that have large, strong target groups [15]. Furthermore, in Iran like many developing countries, some political actors who have appropriate financial resources and facilities, seek to develop short-term and popular policies to pursue their own agenda, e.g. immediate implementation of health transformation plan [44]. Therefore, FPR-related policies, which require long-term views, are challenged.

The results of this study also represented that social network density was low. Thus, increasing the interaction and collaboration among stakeholders must be considered to improve the FPR-related decision- and policy-making processes. This recommendation was supported by other studies [47, 50] in the literature which indicated that if the relevant actors use coherent and coordinated strategies throughout their policy processes, it has the potential to improve the present situation.

### Limitations

This study has confronted with a number of limitations that should be taken into account alongside the results. Despite the efforts of the research team, some samples, especially health policymakers, were reluctant to participate. Further, the findings of this study are context-specific and so it is impossible to apply them directly in other societies. However, considering and identifying the stakeholders can be useful to facilitate the identification of essential stakeholders in various settings.

## Conclusions

The findings of this study showed that there are several stakeholders in FPR-related decision- and policy-making processes in Iran. As many powerful and influential stakeholders had a low interest level to participate in policy processes, using careful and effective strategies, that is, ongoing negotiations, receiving advocacy, and promoting awareness all round among senior managers and policy-makers can be helpful. In addition, street-level actors such as providers, rehabilitation experts, and disabled groups should be taken into consideration in policy-making processes. The results of SNA also represented that social network density was low. Thus, increasing the interaction and collaboration among stakeholders must be considered to improve the FPR-related decision- and policy-making processes. This recommendation was supported by other studies [45, 47] in the literature which indicated that if the relevant actors use coherent and coordinated strategies throughout their policy processes, it has the potential to improve the present situation. Our findings provide lessons for other countries, especially in the EMRO and Middle East and North Africa (MENA) regions, which confront similar issues in the physical rehabilitation sector.

## Data Availability

The datasets generated and/or analyzed during the current study are not publicly available due to the ethical principles of our qualitative study but are available from the corresponding author on reasonable request.

## Abbreviations

CHE: Catastrophic health expenditure
OOP: Out-of-pocket
PRF: Physical rehabilitation financing
SNA: Social Network Analysis
HCHI: High Council of Health Insurance
SSO: Social Security Organization
IHIO: Iran Health Insurance Organization
AFSSO: Armed Forces Social Security Organization
FMVA: Foundation of Martyrs and Veterans Affairs
MOHME: Ministry of Health and Medical education
PBO: Planning and Budget Organization
